# Molecular Characterization of Multidrug-Resistant *Escherichia coli* Isolated from Beef and Chicken Meat Products in Samsun, Türkiye

**DOI:** 10.3390/antibiotics15070668

**Published:** 2026-07-08

**Authors:** Goknur Terzi Gulel, Sibel Kanat, Esra Ekizceli

**Affiliations:** Department of Food Hygiene and Technology, Faculty of Veterinary Medicine, Ondokuz Mayis University, 55139 Samsun, Türkiye; sibel.kanat@omu.edu.tr (S.K.); esrakucukgoz@hotmail.com (E.E.)

**Keywords:** *Escherichia coli*, virulence genes, antimicrobial resistance, multidrug resistance, beef meat, chicken meat

## Abstract

**Background:** Foodborne pathogenic *Escherichia coli* is a major public health concern due to its frequent association with meat and meat products and its potential to harbor virulence factors and antimicrobial resistance (AMR) determinants. **Objective:** This study aimed to investigate the prevalence, virulence gene profiles, and AMR patterns of *E. coli* isolates obtained from beef and chicken meat products. **Methods:** A total of 200 beef and chicken meat product samples were collected from retail markets in Samsun, Türkiye. Isolation of *E. coli* was performed using conventional culture-based methods, and PCR targeting the *uspA* gene was used for molecular confirmation. The presence of virulence genes (*stx1*, *stx2*, *eae*, and *hlyA*) was investigated by PCR. Antimicrobial susceptibility testing was conducted using the disk diffusion method, and multidrug resistance (MDR) and multiple antibiotic resistance (MAR) indices were evaluated. **Results:** Among the 200 samples analyzed, 80 (40%) were positive for *E. coli*, including 38 (38%) beef and 42 (42%) chicken meat samples. A total of 185 *E. coli* isolates were recovered and confirmed by PCR. Virulence gene analysis showed that *stx2* was the most prevalent gene (51.4%), followed by *eae* (37.3%), *hlyA* (13.0%), and *stx1* (6.5%). Antimicrobial susceptibility testing demonstrated high resistance rates to tetracycline (69.7%), ampicillin (58.4%), trimethoprim–sulfamethoxazole (48.1%), streptomycin (40.5%), nalidixic acid (40.0%), chloramphenicol (40.0%), and ciprofloxacin (34.1%). In contrast, the lowest resistance rates were observed for imipenem (2.2%), amoxicillin–clavulanate (4.9%), and amikacin (7.6%). Moreover, 126 isolates (68.1%) were identified as MDR, exhibiting resistance to at least three antimicrobial agents. The MAR index ranged from 0.06 to 1.00. **Conclusions:** The coexistence of virulence-associated genes and high AMR rates among *E. coli* isolates from meat products indicates a potential public health risk. These findings highlight the importance of continuous monitoring of pathogenic and antimicrobial-resistant *E. coli* throughout the food production chain.

## 1. Introduction

Meat is an important component of the human diet due to its high nutritional value, particularly its rich content of proteins, vitamins, and minerals [1]. However, its high water activity, nutrient availability, and near-neutral pH provide favorable conditions for microbial growth, making meat and meat products highly susceptible to spoilage and microbial contamination. During slaughtering, processing, transportation, packaging, and distribution, cross-contamination may occur at multiple stages, thereby increasing the microbial load and compromising food safety [2]. For this reason, meat and meat products are considered an important public health concern with respect to several pathogenic microorganisms, particularly *Salmonella* spp., *Campylobacter jejuni*, *Escherichia coli* (*E. coli*) and *Listeria monocytogenes* [3,4].

*E. coli* is a Gram-negative, facultative anaerobic, non-spore-forming bacterium commonly found in the intestinal microbiota of humans and warm-blooded animals [5]. Contamination of meat products may occur through fecal material during slaughter and processing or through environmental sources and handling practices. Therefore, *E. coli* is considered both an indicator organism and an important foodborne pathogen associated with meat and meat products [4,6]. Raw or undercooked meat products, particularly minced meat and hamburger products, play a major role in the transmission of pathogenic *E. coli* strains to humans [7]. In addition, certain pathogenic strains, especially Shiga toxin-producing *E. coli* (STEC), are capable of causing severe gastrointestinal diseases, including hemorrhagic colitis and hemolytic uremic syndrome (HUS), which may result in life-threatening complications [8].

The pathogenicity of *E. coli* is largely associated with specific virulence factors, particularly the Shiga toxin genes (*stx1* and *stx2*), the adhesion-related intimin gene (*eae*), and the hemolysin gene (*hlyA*) [9,10]. The *stx1* and *stx2* genes encode Shiga toxins that inhibit protein synthesis in host cells and contribute to severe clinical manifestations such as hemorrhagic colitis and HUS [11,12]. The *eae* gene encodes intimin, an outer membrane adhesion protein responsible for intimate attachment to intestinal epithelial cells and the formation of attaching-and-effacing lesions [9]. The *hlyA* gene encodes α-hemolysin, an RTX (repeats-in-toxin) family toxin associated with membrane damage, cell lysis, and increased disease severity [13]. Detection of these virulence-associated genes in meat products is therefore considered an important indicator of potential public health risk.

In recent years, antimicrobial resistance (AMR) has emerged as a major global public health concern, threatening the effectiveness of antimicrobial agents used in both human and veterinary medicine. The extensive and often uncontrolled use of antimicrobials in food-producing animals has contributed substantially to the emergence and dissemination of resistant bacteria throughout the food chain [14,15]. Among foodborne pathogens, *E. coli* is considered a key indicator organism for AMR surveillance because of its widespread distribution and its ability to acquire and transfer resistance determinants through mobile genetic elements. In particular, MDR *E. coli* strains have increasingly been reported in retail meat products worldwide, posing a considerable risk to consumers [16,17,18].

The present study aimed to determine the occurrence of *E. coli* in beef and chicken meat products, characterize the prevalence of major virulence genes (*stx1*, *stx2*, *eae*, and *hlyA*), and evaluate the AMR profiles of the isolates. These findings may contribute to ongoing surveillance programs and support One Health strategies aimed at limiting the dissemination of AMR foodborne pathogens throughout the food production chain.

## 2. Results

### 2.1. Prevalence and Virulence Gene Profiles of E. coli Isolates

*E. coli* was detected in 80 (40%) of the 200 samples analyzed. Of these, 38/100 (38%) beef meat product samples and 42/100 (42%) chicken meat product samples were positive for *E. coli*. Among beef meat products, the highest contamination rate was observed in hamburger samples (13/20, 65%), followed by minced beef (11/20, 55%) and meatballs (8/20, 40%), whereas diced beef and sausage showed lower contamination levels (3/20, 15% each). Among chicken meat products, chicken breast (15/20, 75%) and chicken drumstick (13/20, 65%) exhibited the highest contamination rates, followed by chicken wing (11/20, 55%) and chicken sausage (3/20, 15%), while no *E. coli* was detected in chicken burger samples. No statistically significant difference was observed in *E. coli* prevalence between beef and chicken meat products (χ^2^ = 0.33, *p* = 0.56).

**Figure 1:**
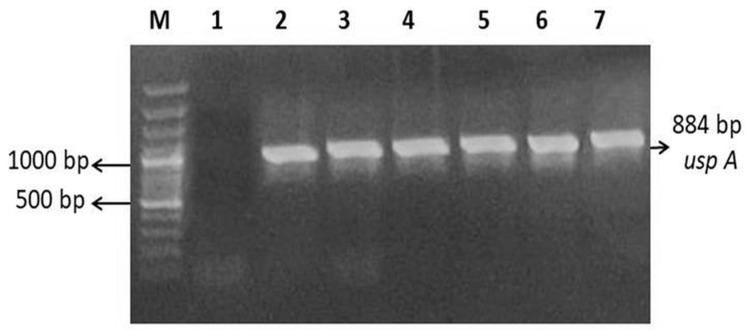
PCR amplification of the *uspA* gene in *E. coli* isolates. M: 100 bp DNA marker; Lane 1: negative control (no template), Lane 2: positive control (*E. coli* ATCC 43888); Lanes 3–7: *uspA*-positive *E. coli* isolates obtained from beef and chicken meat products (isolates 2a, 9a, 14a, 28b and 43c), respectively.

**Figure 2:**
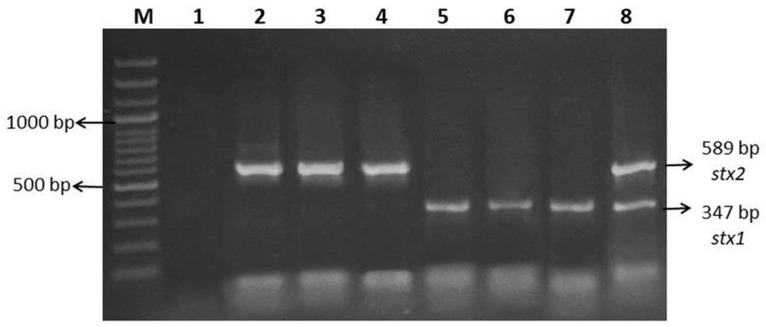
PCR amplification of the *stx1* and *stx2* genes in *E. coli* isolates. M: 100 bp DNA ladder; Lane 1: negative control (no template DNA); Lane 2: positive control (*E. coli* ATCC 43889, *stx2*-positive); Lanes 3–4: *stx2*-positive *E. coli* isolates obtained from minced beef (isolate 14a) and meatball samples (isolate 9a), respectively; Lane 5: positive control (*E. coli* ATCC 43890, *stx1*-positive); Lanes 6–7: *stx1*-positive *E. coli* isolates obtained from hamburger (isolate 73b) and sausage samples (isolate 76a), respectively; Lane 8: *stx1/stx2*-positive *E. coli* isolate obtained from a chicken breast sample (isolate 28b).

**Figure 3:**
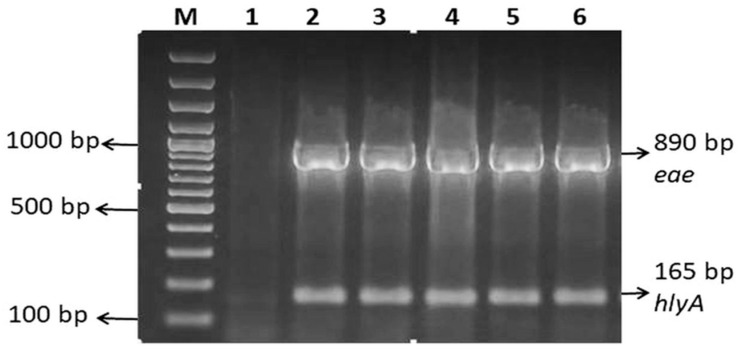
PCR amplification of the *eae* and *hlyA* genes in *E. coli* isolates. M: 100 bp DNA marker; Lane 1: negative control (no template), Lane 2: positive control (*E. coli* O157:H7 ATCC 43888, *hlyA*+, *eae*+); Lanes 3–6: *eae* and *hlyA*-positive *E. coli* isolates obtained from chicken wing (isolate 19a), meatball (isolate 61a), hamburger (isolate 66a) and diced beef samples (isolate 43c), respectively.

The prevalence and virulence profiles of the *E. coli* isolates are summarized in Table 1 and Table 2. A total of 185 *E. coli* isolates were recovered and confirmed by PCR targeting the *uspA* gene. The expected amplicon sizes were 884 bp for *uspA*, 347 bp for *stx1*, 589 bp for *stx2*, 890 bp for *eae*, and 165 bp for *hlyA* (Figure 1, Figure 2 and Figure 3). Virulence gene analysis revealed that 12/185 (6.5%) isolates carried *stx1*, 95/185 (51.4%) carried *stx2*, 69/185 (37.3%) carried *eae*, and 24/185 (13.0%) carried *hlyA*. Among the investigated virulence genes, *stx2* was the most prevalent, followed by *eae*, *hlyA*, and *stx1*. No statistically significant differences were observed between beef and chicken isolates regarding *stx1*, *stx2*, and *eae* genes (*p* > 0.05), while *hlyA* showed a significantly higher prevalence in beef isolates (*p* < 0.001). Co-occurrence analysis of virulence genes revealed multiple combination patterns among the *E. coli* isolates (Table 3). The most prevalent combination was *stx2 + eae*, detected in 43 (23.2%) isolates, including 24 (25.8%) beef-derived and 19 (20.7%) chicken-derived isolates.

The distribution of virulence genes according to MDR status in beef and chicken isolates is presented in Table 4. In chicken-derived isolates, MDR prevalence was notably high among *stx2*-positive (93.8%) and *eae*-positive (92.1%) isolates. In beef isolates, MDR prevalence was more evenly distributed across virulence gene profiles. Overall, *stx2*-positive isolates showed a higher proportion of MDR (70.5%) compared to other virulence genes. However, variability in MDR distribution among virulence gene profiles was observed, indicating heterogeneous resistance patterns across different genotypes and meat sources.

### 2.2. Antimicrobial Resistance and Multidrug Resistance Profiles of E. coli Isolates

**Figure 4:**
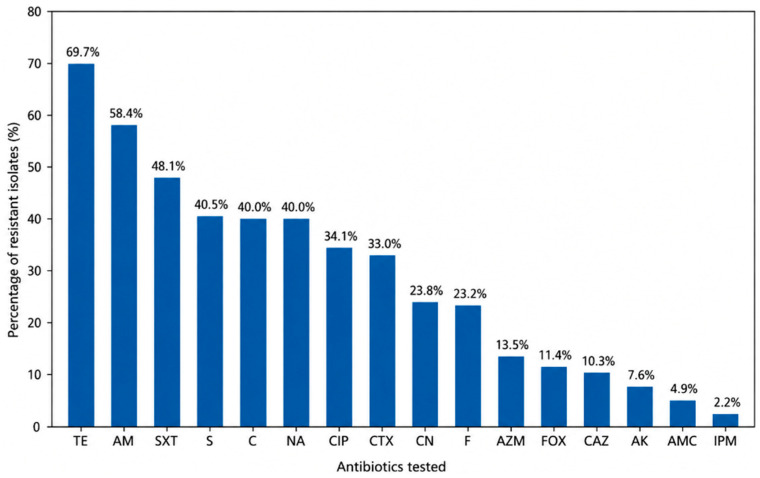
Antimicrobial resistance rate of *E. coli* isolates (*n* = 185). TE, Tetracycline; AM, Ampicillin; SXT, Trimethoprim–Sulfamethoxazole; S, Streptomycin; C, Chloramphenicol; NA, Nalidixic Acid; CIP, Ciprofloxacin; CTX, Cefotaxime; CN, Gentamicin; F, Nitrofurantoin; AZM, Azithromycin; FOX, Cefoxitin; CAZ, Ceftazidime; AK, Amikacin; AMC, Amoxicillin–Clavulanic Acid; IPM, Imipenem.

AMR profiles of *E. coli* isolates are summarized in Table 5 and Figure 4. High resistance rates were observed for tetracycline (69.7%), ampicillin (58.4%), trimethoprim–sulfamethoxazole (48.1%), streptomycin (40.5%), nalidixic acid (40.0%), and chloramphenicol (40.0%). Moderate resistance was observed for ciprofloxacin (34.1%), whereas low resistance rates were detected for amikacin (7.6%), amoxicillin–clavulanate (4.9%), and imipenem (2.2%).

The multiple antibiotic resistance (MAR) index values and distribution patterns are presented in Table 6. The MAR index was calculated as MAR = a/b, where *a* represents the number of antibiotics to which an isolate is resistant and *b* the total number tested. The MAR index ranged from 0.06 to 1.00, with an overall mean value of 0.285 ± 0.22. Significant differences were observed between meat types, with higher MAR index values in chicken isolates (0.406 ± 0.18) compared to beef isolates (0.165 ± 0.19) (Mann–Whitney U test, *p* < 0.001). Of the 185 isolates, 113 (61.1%) exhibited MAR values > 0.2, while 72 (38.9%) rall, 126 isolates (68.1%) were classified as MDR, defined as resistance to at least three antimicrobial agents. Resistance pattern analysis demonstrated a wide distribution of resistance phenotypes among the isolates. The proportions of isolates resistant to 1, 2, 3, 4, 5, 6, 7, 8, 9, 10, 11, 12, and 16 antibiotics were 9.7%, 4.8%, 7.0%, 9.1%, 15.1%, 8.1%, 5.9%, 5.4%, 7.5%, 4.8%, 3.2%, 1.1%, and 0.5%, respectively. Notably, a considerable proportion of isolates exhibited resistance to six or more antibiotics, and one isolate showed resistance to all tested antimicrobials.

Representative positive isolates are shown; all 185 isolates were analyzed.

## 3. Discussion

### 3.1. E. coli Prevalence in Red Meat and Poultry

*E. coli* is recognized as one of the most important foodborne pathogens, with certain pathogenic strains capable of causing severe and potentially life-threatening illnesses in humans. Therefore, its presence in meat and meat products represents a significant concern for both food safety and public health. In the present study, *E. coli* was detected in 42% of poultry meat and poultry products and 38% of red meat and meat samples, with no statistically significant difference between the two groups (χ^2^ = 0.33, *p* = 0.56). The prevalence observed in red meat indicates a considerable level of contamination at the retail level and highlights its potential role as a vehicle for foodborne transmission.

Previous studies have consistently reported the occurrence of *E. coli* in retail red meat products, although prevalence rates vary considerably among regions and production systems, including studies conducted in Egypt, Senegal, Vietnam, and other African countries [19,20,21,22,23]. Reported prevalence values ranging from 3% to 62% reflect substantial variability, which is strongly influenced by slaughter hygiene, carcass handling, processing infrastructure, and retail-level sanitation practices. These findings emphasize the critical role of regional food safety systems and production standards in determining contamination levels.

In poultry products, contamination levels observed in the present study were consistent with findings reported in studies conducted in Pakistan, the United States (Washington, D.C. and Southern California), Iran, Egypt, and several European countries, where the prevalence of *E. coli* in poultry meat has been reported to range from 8% to 68% [23,24,25,26,27]. Poultry is widely recognized as a major reservoir of *E. coli* due to its high intestinal bacterial load and intensive processing conditions during slaughter, evisceration, and distribution. This variability suggests that contamination is influenced by multiple factors along the production chain, including farm-level hygiene, slaughtering practices, processing environments, and retail handling.

Overall, the present study demonstrated a higher prevalence of *E. coli* in poultry meat compared to red meat, which is consistent with previous investigations [24,28]. Poultry generally harbors a higher intestinal bacterial load, increasing the likelihood of contamination during slaughter, particularly during evisceration. In addition, high-speed processing lines and extensive handling during poultry processing may facilitate cross-contamination between carcasses. Furthermore, the structural characteristics of poultry skin provide a favorable surface for bacterial attachment and persistence. The combination of these factors may promote the dissemination of *E. coli* throughout the processing chain and contribute to the higher contamination levels observed in poultry products. In contrast, contamination in red meat is often more localized to surface tissues and may occur at lower levels under relatively more controlled processing conditions.

Among red meat samples, the highest contamination rate was observed in hamburgers (65%), followed by minced beef (55%) and meatballs (40%), whereas diced beef and sausage showed considerably lower contamination levels (15%). In poultry meat samples, chicken breast (75%) and drumstick (65%) exhibited the highest contamination rates, followed by chicken wing (55%). In contrast, lower contamination levels were detected in chicken sausage (15%), while *E. coli* was not detected in chicken burger samples. These findings indicate that contamination levels may vary not only between meat species but also according to product type and processing characteristics. Comparable patterns have also been reported in previous studies, in which minced and processed meat products were identified as important sources of *E. coli* contamination [19].

These findings demonstrate notable differences not only between meat types but also among product categories. The higher contamination rates observed in comminuted meat products, such as minced beef and hamburgers, may be attributed to increased surface area, disruption of muscle structure, and a greater risk of cross-contamination during processing. Grinding disrupts the natural protective barriers of whole-muscle meat and facilitates the distribution of microorganisms throughout the product, resulting in a more homogeneous contamination pattern, even when initial bacterial loads are low. In addition, repeated handling, mixing processes, and contact with processing equipment may increase opportunities for microbial dissemination. Similar observations regarding the increased susceptibility of minced and processed meat products to *E. coli* contamination have also been reported previously, including the detection of *E. coli* O157 in minced beef and beef burger products [7]. Collectively, these factors reinforce the role of minced and processed meat products as high-risk matrices for foodborne pathogens such as *E. coli*.

In addition, inadequate storage conditions, including temperature abuse and interruptions in the cold chain, may further contribute to bacterial survival and proliferation in poultry meat products. Insufficient hygiene practices during processing and handling may also play a critical role in the persistence and spread of *E. coli* contamination. To reduce this public health risk, the implementation of strict food safety management systems is essential. This includes regular hygiene training for personnel, proper application of Hazard Analysis and Critical Control Point (HACCP) principles, effective sanitation of equipment and processing environments, and strict maintenance of the cold chain during storage and distribution. Strengthening these control measures can significantly reduce the risk of *E. coli* contamination and improve overall food safety.

A limitation of this study is that samples were collected from a single geographic region (Samsun, Türkiye), which may limit the generalizability of the findings to other regions of Türkiye. In addition, no molecular typing or clonal relatedness analysis was performed; therefore, isolates cannot be confirmed as genetically distinct strains and should be interpreted as phenotypically selected colonies. Furthermore, molecular characterization of AMR determinants was not conducted, and the genetic basis of the observed resistance phenotypes could not be determined. Future studies incorporating resistance gene profiling, molecular typing, and broader geographical sampling would provide a more comprehensive understanding of the epidemiology and public health significance of these isolates.

### 3.2. Virulence Gene Profiles of E. coli Isolates

The detection of virulence-associated genes in *E. coli* isolates recovered from retail meat products provides important insight into their pathogenic potential and public health significance. In the present study, *stx2* was the most prevalent gene (51.4%), followed by *eae* (37.3%), *hlyA* (13.0%), and *stx1* (6.5%). The predominance of *stx2* is particularly important because this gene is strongly associated with severe clinical outcomes such as hemorrhagic colitis and hemolytic uremic syndrome (HUS) [10]. Similar findings have been reported by Zeinali et al. [27], who also observed *stx2* as the dominant virulence gene in chicken meat isolates, suggesting a consistent epidemiological pattern across food sources.

*stx2*-positive STEC strains have been reported to be more frequently associated with severe human diseases than strains carrying only *stx1*. Therefore, the high prevalence of *stx2* may indicate the circulation of *E. coli* populations with enhanced pathogenic potential within the meat production chain. In addition, Shiga toxin-encoding bacteriophages play a crucial role in the horizontal transfer of virulence determinants, facilitating the dissemination and persistence of highly virulent genotypes in food-producing animals and retail meat products.

The *eae* gene, encoding the adhesion protein intimin, was detected in 37.3% of the isolates, indicating a considerable proportion of strains with enhanced colonization potential. This prevalence was higher than those reported in several previous studies, including Zeinali et al. [27], who conducted a study in Iran, and Fatima et al. [26], who reported relatively low *eae* positivity rates (7–11%) in poultry-associated isolates from Pakistan. In contrast, Thierry et al. [29] reported a higher prevalence of *eae*-positive STEC isolates (47.6%) in animal-derived food sources from Mauritius, indicating variability in adhesion-associated virulence profiles across different studies. Since intimin plays a critical role in bacterial attachment to intestinal epithelial cells, *eae*-positive isolates may have an increased ability to establish infection and persist within the host. These differences may be attributed to variations in animal sources, geographic regions, and production practices.

Co-occurrence analysis revealed the presence of *stx2* + *eae* in a substantial proportion of isolates, which is of particular concern due to its strong association with enterohemorrhagic *E. coli* (EHEC) pathotypes. Notably, *stx2* + *eae* was the most frequent co-occurring virulence profile (23.2%), particularly in beef-derived isolates. Strains harboring both genes possess an enhanced ability to adhere to intestinal epithelium and produce Shiga toxins, thereby increasing their pathogenic potential. This combination of virulence factors may therefore represent a greater public health risk than isolates carrying only a single virulence determinant.

The *hlyA* gene, encoding enterohemolysin, was detected in 13.0% of the isolates, with a higher frequency observed in beef-derived samples. This finding may suggest a greater contribution of red meat products to the dissemination of hemolysin-producing *E. coli*. Enterohemolysin is considered an accessory virulence factor that may act synergistically with Shiga toxins and adhesion factors, potentially contributing to increased tissue damage and disease severity. Similar prevalence rates of *hlyA*-positive *E. coli* isolates from retail meat products have been reported by Martínez-Vázquez et al. [30].

The present study investigated the association between virulence gene carriage and MDR phenotype in *E. coli* isolates. A higher proportion of MDR was observed among *stx2* and *eae* positive isolates, particularly in chicken-derived samples, where MDR rates exceeded 90%. This finding suggests that poultry-associated *E. coli* strains may serve as important reservoirs for both virulence and AMR determinants. However, the distribution was not uniform across all genes, indicating that virulence and resistance traits may be partially independent. These results are consistent with previous reports indicating that AMR and virulence determinants are often carried on different mobile genetic elements, leading to heterogeneous associations in foodborne *E. coli* populations.

### 3.3. Antimicrobial Resistance

The emergence and dissemination of AMR *E. coli* in foods of animal origin represent a major public health concern worldwide because resistant strains may be transmitted to humans through the food chain. In the present study, high resistance rates were observed particularly for tetracycline (69.7%), ampicillin (58.3%), trimethoprim–sulfamethoxazole (48.1%), streptomycin (40.5%), nalidixic acid (40.0%), and chloramphenicol (40.0%). Similar resistance patterns have been reported in previous studies investigating meat-derived *E. coli* isolates [31,32,33]. The high prevalence of resistance to these commonly used antimicrobials is likely driven by their extensive and long-term use in food animal production systems, which exerts selective pressure and promotes the persistence of resistant bacterial populations.

In particular, the high resistance to tetracycline and ampicillin may reflect the long-term and extensive use of these antimicrobial classes in animal husbandry. Tetracycline resistance is frequently mediated by transferable *tet* genes, whereas resistance to β-lactam antibiotics such as ampicillin is commonly associated with plasmid-mediated β-lactamase production. However, molecular detection of β-lactamase genes was not performed in the present study, which represents a limitation. The dissemination of mobile genetic elements may facilitate the co-selection and accumulation of multiple resistance determinants within bacterial populations. Future studies incorporating molecular characterization of resistance genes are therefore warranted to better elucidate the genetic basis of AMR.

The present study also demonstrated a high prevalence of MDR (68.1%), which was higher than the MDR rates reported in several previous studies [31,32]. In addition, the MAR index ranged from 0.06 to 1.00, indicating the circulation of isolates with diverse resistance profiles. Notably, one isolate exhibited resistance to all tested antimicrobials, highlighting the potential public health risk associated with highly resistant *E. coli* strains in retail meat products. The occurrence of isolates resistant to multiple antimicrobial classes may indicate sustained antimicrobial selection pressure and suggests that resistant bacteria and resistance genes may persist and disseminate throughout the food production chain. The higher MAR burden in poultry-derived isolates was statistically significant (*p* < 0.001), further supporting the role of chicken meat as a potential reservoir of MDR *E. coli* of public health concern.

Resistance to critically important antimicrobials such as ciprofloxacin was also considerable (34.1%) in the present study. Similar findings have been reported in retail meat isolates worldwide, although resistance levels vary depending on regional antimicrobial usage practices, farm management systems, and regulatory policies [23,32]. In Türkiye, Sahin et al. [34] reported the circulation of ESBL-producing and MDR *E. coli* isolates in poultry, while Dishan et al. [35] demonstrated high levels of AMR and virulence-associated characteristics among STEC isolates recovered from retail chicken meat. These findings further support the role of poultry-associated food products as important reservoirs for AMR determinants and potentially pathogenic *E. coli* strains. The widespread occurrence of MDR *E. coli* in meat products further supports concerns regarding the role of retail foods as reservoirs for AMR determinants.

In addition to antimicrobial selection pressure, differences in the physiological environments of poultry and cattle gastrointestinal tracts may contribute to the observed variation in AMR patterns among meat-derived *E. coli* isolates. In ruminants, the presence of multiple stomach compartments, particularly the rumen with an acidic environment (pH approximately 5), may impose distinct selective pressures on bacterial populations compared with the avian intestinal tract. These conditions may influence bacterial survival and stress adaptation, thereby shaping the composition of *E. coli* populations entering the food chain. In support of this concept, Zhang et al. [36] demonstrated that the global regulators RpoS and Crp coordinate acid stress responses in *E. coli* by regulating metabolic pathways, membrane-associated functions, and stress adaptation under acidic conditions.

The emergence of MDR *E. coli* in retail meat products may therefore involve not only the acquisition of AMR determinants but also enhanced bacterial fitness under selective environmental pressures. The interplay between stress response systems and AMR may promote the persistence and dissemination of MDR strains through potential co-selection mechanisms. However, the mechanistic relationship between acid tolerance and AMR remains incompletely understood. Further studies investigating stress response phenotypes in MDR isolates would provide valuable insights into their ecological success in food production chains.

## 4. Materials and Methods

### 4.1. Sample Collection

A total of 200 retail meat samples, including 100 beef products (diced beef, minced beef, meatballs, hamburgers, and sausages) and 100 chicken products (chicken breast, drumsticks, wings, sausages, and burgers), were collected from various retail outlets, local butcher shops, and supermarket chains in Samsun, Türkiye, between November 2024 and February 2025. Beef products were obtained from 11 local butcher shops and two national supermarket chains, while chicken products were collected from seven local poultry retailers, local butcher shops, and supermarket chains operating in the study area. All samples consisted of retail meat products; no live animals were included in this study. Samples reflect locally available products within the regional retail market and may represent meats distributed through local and regional supply chains. All samples were transported to the laboratory under cold-chain conditions and processed promptly upon arrival.

The sample size was determined based on feasibility, availability of retail meat products, and in accordance with previously published similar studies. Samsun was selected as the study area due to its status as the largest province in the Middle Black Sea Region and its role as a major regional center for meat production and food distribution. Information regarding the farm of origin, slaughterhouse, production region, or supply chain of the animals was not available for the sampled products. Therefore, the results should be interpreted as representative of retail meat products marketed in Samsun rather than specific livestock production systems or geographical regions.

### 4.2. Isolation and Characterization of E. coli

The presence of *E. coli* in meat and meat products was determined using conventional culture-based methods. For analysis, 10 g of each food sample was homogenized with 90 mL of Maximum Recovery Diluent (Merck, Darmstadt, Germany) using a stomacher for 2 min. Subsequently, 0.1 mL aliquots of the homogenate were spread onto Violet Red Bile Agar (VRBA; Merck, Germany) and incubated at 37 °C for 24 h for coliform enumeration. Red–pink colonies on VRBA were transferred to Eosin Methylene Blue (EMB) agar (Merck, Germany) and incubated at 37 °C for 24 h. Presumptive colonies exhibiting a metallic sheen on EMB agar were subcultured on Tryptic Soy Agar (TSA; Merck, Germany) and incubated at 37 °C for 18–24 h for biochemical confirmation. Colonies showing typical and/or distinct morphological characteristics were considered for further analysis. From each positive sample, 2–3 confirmed *E. coli* isolates were randomly selected from morphologically distinct colonies to ensure balanced representation and to avoid overrepresentation of any individual sample. Presumptive *E. coli* isolates were preserved in cryovials containing Tryptic Soy Broth (TSB) supplemented with 20% glycerol and stored at −20 °C [37,38].

### 4.3. DNA Extraction

Genomic DNA from bacterial isolates was extracted using the boiling method, a rapid and cost-effective DNA preparation approach previously used for PCR-based detection of bacterial virulence genes. The boiling method was selected because it provides DNA of sufficient quality for PCR-based molecular analyses and has been successfully applied in previous studies for the detection of bacterial pathogens [39]. Isolates stored at −20 °C were first revived in Tryptic Soy Broth (TSB; Merck, Darmstadt, Germany) and incubated at 37 °C for 18–24 h. The cultures were then streaked onto Eosin Methylene Blue (EMB) agar, and colonies showing a metallic sheen were subcultured on Tryptic Soy Agar (TSA; Merck, Darmstadt, Germany) and incubated at 37 °C for 18–24 h. The obtained colonies were suspended in 200 µL of sterile deionized water in Eppendorf tubes. Denaturation was performed at 95 °C for 10 min using a dry block heater (Biosan TDB-120, Biosan, Riga, Lithuania). The samples were then centrifuged at 10,000× *g* for 5 min at 4 °C (Hettich Universal 320R, Tuttlingen, Germany). The supernatant was transferred to a sterile Eppendorf tube and stored at −20 °C until molecular analysis.

The quality and purity of the extracted genomic DNA were assessed using a spectrophotometer by determining the A260/A280 absorbance ratio. Additionally, DNA integrity was checked by 1.5% agarose gel electrophoresis and visualized under UV illumination. The suitability of the extracted DNA for downstream PCR applications was further confirmed by amplification of the *uspA* gene, which served as a species-specific internal control marker for the identification of *E. coli*.

### 4.4. PCR Confirmation of E. coli Isolates Using the uspA Gene

Presumptive *E. coli* colonies were confirmed by PCR targeting the *uspA* gene. All primers used in this study are listed in Table 7. The PCR mixture was prepared in a final volume of 25 µL containing 1× PCR buffer (500 mM KCl, 200 mM Tris–HCl), 0.2 mM dNTPs, 2 mM MgCl_2_, 0.5 µM of each primer, 1 U Taq DNA polymerase, and 2 µL of template DNA. PCR reactions were performed using a thermal cycler (MJ Mini, PTC-1148; Bio-Rad Laboratories, Hercules, CA, USA). The amplification conditions consisted of an initial denaturation at 94 °C for 5 min, followed by 32 cycles of denaturation at 94 °C for 30 s, annealing at 63 °C for 30 s, and extension at 72 °C for 1.5 min, with a final extension at 72 °C for 5 min.

### 4.5. Detection of Virulence Genes in E. coli Isolates by PCR

PCR assays were performed for the detection of virulence genes (*stx1*, *stx2*, *hlyA*, and *eae*) in *E. coli* isolates. The PCR mixture was prepared in a final volume of 25 µL containing 1× PCR buffer (500 mM KCl, 200 mM Tris–HCl), 0.2 mM dNTPs, 2 mM MgCl_2_, 0.8 µM of each primer, 1 U Taq DNA polymerase, and 2 µL of template DNA. The amplification conditions consisted of an initial denaturation at 95 °C for 5 min, followed by 35 cycles of denaturation at 95 °C for 30 s, annealing at 57 °C for 45 s for *stx1* and *stx2* genes or 55 °C for 45 s for *hlyA* and *eae* genes, and extension at 72 °C for 45 s, with a final extension at 72 °C for 7 min. All PCR assays were performed in duplicate to ensure reproducibility of the results.

### 4.6. Agarose Gel Electrophoresis

PCR products were analyzed on 1.5% agarose gel prepared in 1× TBE buffer (Tris–borate–EDTA; 89 mM Tris, 89 mM boric acid, 2 mM EDTA, pH 8.3) and stained with 0.5 µg/mL ethidium bromide. Electrophoresis was carried out at 80 V for 1 h using a horizontal electrophoresis system (multiSUB Horizontal System, Cleaver Scientific, Rugby, UK). The *uspA* gene was visualized at 884 bp, *stx1* at 347 bp, *stx2* at 589 bp, *eae* at 890 bp, and *hlyA* at 165 bp under a UV transilluminator (Wise-UV WUV-L50, DAIHAN Scientific, Seoul, Republic of Korea).

### 4.7. Antimicrobial Susceptibility Testing

Antimicrobial susceptibility of the *E. coli* isolates against various antibiotics was determined using the disk diffusion method according to the guidelines of the Clinical and Laboratory Standards Institute (CLSI) [44,45]. The antibiotic panel was selected to represent major antimicrobial classes commonly used in human and veterinary medicine, covering different mechanisms of action and clinically relevant drugs for the treatment of *E. coli* infections. The selection was also consistent with widely used antimicrobial susceptibility testing panels and the WHO Critically Important Antimicrobials list. The antibiotics used in this study are listed in Table 5. For this purpose, *E. coli* isolates were grown in Mueller–Hinton Broth (MHB; Merck, Darmstadt, Germany) at 35 °C for 18–24 h. After incubation, fresh cultures were adjusted to a turbidity equivalent to 0.5 McFarland standard (~1 × 10^8^ CFU/mL) using a densitometer (DEN-1, Biosan, Riga, Latvia). Then, 100 µL of the bacterial suspension was uniformly spread on Mueller–Hinton Agar (MHA; Merck, Darmstadt, Germany) plates using a sterile cotton swab and allowed to dry for 15 min. Antibiotic discs (Bioanalyse, Ankara, Turkey) were then placed on the agar surface, and the plates were incubated at 35 °C for 18–24 h. Following incubation, the diameters of inhibition zones around each disc were measured. The results were interpreted as susceptible, intermediate, or resistant according to CLSI-recommended breakpoints [46]. *E. coli* ATCC 25922 was used as a quality control strain to ensure the reliability and accuracy of the disk diffusion method, and all results were within the CLSI-recommended quality control ranges.

### 4.8. Multidrug Resistance (MDR) and Multiple Antibiotic Resistance (MAR) Index

Isolates showing resistance to at least one agent in three or more antimicrobial classes were classified as MDR according to the criteria proposed by Magiorakos et al. [47]. The multiple antibiotic resistance (MAR) index for each isolate was calculated using the following formula: MAR index = a/b, where *a* represents the number of antibiotics to which the isolate was resistant and *b* represents the total number of antibiotics tested [48]. High MAR index values were considered indicative of exposure to environments with frequent antimicrobial use.

### 4.9. Statistical Analysis

Statistical analyses were performed using SPSS Statistics software (Version 22.0; IBM Corp., Armonk, NY, USA). Differences in the prevalence of *E. coli* isolates, AMR patterns, and virulence gene frequencies between beef and chicken samples were evaluated using Pearson’s Chi-square test. Comparisons of MAR index values between beef- and chicken-derived isolates were performed using the Mann–Whitney U test. A *p*-value of <0.05 was considered statistically significant.

## 5. Conclusions

In conclusion, the present study demonstrated that retail beef and chicken meat products may serve as important reservoirs of *E. coli* harboring virulence determinants and MDR traits. The coexistence of virulence-associated genes and AMR determinants among the isolates increases the potential public health risk associated with contaminated meat products. Therefore, continuous surveillance programs, prudent antimicrobial use in food animal production, and improved hygienic practices during slaughtering and meat processing remain essential for limiting the dissemination of AMR *E. coli* throughout the food chain. Furthermore, additional molecular characterization of resistance and virulence determinants, particularly through whole-genome sequencing-based studies, may provide deeper insight into the epidemiology and transmission dynamics of pathogenic *E. coli* strains associated with retail meat products.

## Figures and Tables

**Table 1:** Prevalence of *E. coli* in beef and chicken meat products.

Type of Sample	No. of Samples Examined (*n*)	Positive Sample *n* (%)	No. of *E. coli* (+) Isolates
Beef meat products			
Diced beef	20	3 (15%)	11
Minced beef	20	11 (55%)	21
Meatball	20	8 (40%)	20
Hamburger	20	13 (65%)	34
Sausage	20	3 (15%)	7
Total beef	100	38 (38%)	93
Chicken meat products			
Breast	20	15 (75%)	30
Drumstick	20	13 (65%)	33
Wing	20	11 (55%)	26
Sausage	20	3 (15%)	3
Burger	20	ND	ND
Total chicken	100	42 (42%)	92
Overall total	200	80 (40%)	185

ND: not detected, (+): Positive.

**Table 2:** Distribution of virulence genes among *E. coli* isolates recovered from beef and chicken meat products.

Type of Sample	No. of *E. coli* *uspA* (+) Isolates	*stx1* *n* (%)	*stx2* *n* (%)	*eae* *n* (%)	*hlyA* *n* (%)
Beef meat products					
Diced beef	11	0 (0.0%)	3 (27.3%)	3 (27.3%)	1 (9.1%)
Minced beef	21	5 (23.8%)	10 (47.6%)	7 (33.3%)	6 (28.6%)
Meatball	20	1 (5.0%)	15 (75.0%)	7 (35.0%)	6 (30.0%)
Hamburger	34	2 (5.9%)	12 (35.3%)	14 (41.2%)	1 (2.9%)
Sausage	7	1 (14.3%)	7 (100.0%)	0 (0.0%)	6 (85.7%)
Total beef	93	9 (9.7%)	47 (50.5%)	31 (33.3%)	20 (21.5%)
Chicken meat products					
Breast	30	0 (0.0%)	16 (53.3%)	14 (46.7%)	1 (3.3%)
Drumstick	33	1 (3.0%)	16 (48.5%)	12 (36.4%)	1 (3.0%)
Wing	26	2 (7.7%)	14 (53.8%)	9 (34.6%)	2 (7.7%)
Sausage	3	0 (0.0%)	2 (66.7%)	3 (100.0%)	0 (0.0%)
Burger	ND	ND	ND	ND	ND
Total chicken	92	3 (3.3%)	48 (52.2%)	38 (41.3%)	4 (4.3%)
Overall total	185	12 (6.5%)	95 (51.4%)	69 (37.3%)	24 (13.0%)

ND: not detected, (+): Positive.

**Table 3:** Selected virulence gene co-occurrence profiles among *E. coli* isolates.

Virulence Gene Combination	Beef Meat Products *n* (%) (*n* = 93)	Chicken Meat Products *n* (%) (*n* = 92)	Total Sample *n* (%) (*n* = 185)
*stx2* + *eae*	24 (25.8%)	19 (20.7%)	43 (23.2%)
*stx2* + *eae* + *hlyA*	4 (4.3%)	0 (0%)	4 (2.2%)
*stx2* + *hlyA*	15 (16.1%)	3 (3.3%)	18 (9.7%)
*stx1* + *stx2*	8 (8.6%)	2 (2.2%)	10 (5.4%)
*stx1* + *stx2* + *eae* + *hlyA*	2 (2.1%)	0 (0%)	2 (1.1%)
*stx1* + *stx2* + *eae*	4 (4.3%)	1 (1.1%)	5 (2.7%)
*eae* + *hlyA*	4 (4.3%)	1 (1.1%)	5 (2.7%)

**Table 4:** Distribution of virulence genes according to MDR status in beef and chicken *E. coli* isolates.

Virulence Gene	Beef Meat Products		Chicken Meat Products		Total	
	MDR (+)	MDR (−)	MDR (+)	MDR (−)	MDR (+)	MDR (−)
*stx1* (*n* = 12)	5 (55.6%)	4 (44.4%)	2 (66.7%)	1 (33.3%)	7 (58.3%)	5 (41.7%)
*stx2* (*n* = 95)	22 (46.8%)	25 (53.2%)	45 (93.8%)	3 (6.3%)	67 (70.5%)	28 (29.5%)
*eae* (*n* = 69)	12 (38.7%)	19 (61.3%)	35 (92.1%)	3 (7.9%)	47 (68.1%)	22 (31.9%)
*hlyA* (*n* = 24)	10 (50.0%)	10 (50.0%)	4 (100.0%)	0 (0.0%)	14 (58.3%)	10 (41.7%)

(+): Positive; (−): Negative.

**Table 5:** Antibiotic resistance profiles of *E. coli* isolates (*n* = 185).

	No. (%) of *E. coli* Isolates									
Antibiotics	Beef Samples (*n* = 93)			Chicken Samples (*n* = 92)			Total Samples (*n* = 185)			
(Disk Content)	R	I	S	R	I	S	R	I	S	*p*-Value
AK (30 µg)	11 (11.8%)	1 (1.1%)	81 (87.1%)	3 (3.3%)	0 (0.0%)	89 (96.7%)	14 (7.6%)	1 (0.5%)	170 (91.9%)	0.051
AM (10 µg)	28 (30.1%)	10 (10.8%)	55 (59.1%)	80 (87.0%)	4 (4.3%)	8 (8.7%)	108 (58.4%)	14 (7.6%)	63 (34.1%)	<0.001
AMC (20 + 10 μg)	3 (3.2%)	10 (10.8%)	80 (86.0%)	6 (6.5%)	18 (19.6%)	68 (73.9%)	9 (4.9%)	28 (15.1%)	148 (80.0%)	0.119
AZM (15 µg)	2 (2.2%)	12 (12.9%)	79 (84.9%)	23 (25.0%)	6 (6.5%)	63 (68.5%)	25 (13.5%)	18 (9.7%)	142 (76.8%)	<0.001
CAZ (30 µg)	10 (10.8%)	8 (8.6%)	75 (80.6%)	9 (9.8%)	24 (26.1%)	59 (64.1%)	19 (10.3%)	32 (17.3%)	134 (72.4%)	0.007
FOX (30 µg)	16 (17.2%)	2 (2.2%)	75 (80.6%)	5 (5.4%)	6 (6.5%)	81 (88.0%)	21 (11.4%)	8 (4.3%)	156 (84.3%)	0.018
CTX (30 µg)	15 (16.1%)	2 (2.2%)	76 (81.7%)	46 (50.0%)	6 (6.5%)	40 (43.4%)	61 (33.0%)	8 (4.3%)	116 (62.7%)	<0.001
CIP (5 µg)	14 (15.1%)	23 (24.7%)	56 (60.2%)	49 (53.3%)	34 (37.0%)	9 (9.8%)	63 (34.1%)	57 (30.8%)	65 (35.1%)	<0.001
C (30 µg)	22 (23.7%)	3 (3.2%)	68 (73.1%)	52 (56.5%)	16 (17.4%)	24 (26.1%)	74 (40.0%)	19 (10.3%)	92 (49.7%)	<0.001
CN (10 µg)	12 (12.9%)	0 (0.0%)	81 (87.1%)	32 (34.8%)	4 (4.3%)	56 (60.9%)	44 (23.8%)	4 (2.2%)	137 (74.1%)	<0.001
IPM (10 µg)	1 (1.1%)	2 (2.2%)	90 (96.8%)	3 (3.3%)	2 (2.2%)	87 (94.6%)	4 (2.2%)	4 (2.2%)	177 (95.7%)	0.593
NA (30 µg)	17 (18.3%)	0 (0.0%)	76 (81.7%)	57 (62.0%)	19 (20.7%)	16 (17.4%)	74 (40.0%)	19 (10.3%)	92 (49.7%)	<0.001
F (300 µg)	13 (14.0%)	5 (5.4%)	75 (80.6%)	30 (32.6%)	8 (8.7%)	54 (58.7%)	43 (23.2%)	13 (7.0%)	129 (69.7%)	0.005
TE (30 µg)	47 (50.5%)	0 (0.0%)	46 (49.5%)	82 (89.1%)	2 (2.2%)	10 (10.9%)	129 (69.7%)	2 (1.1%)	56 (30.3%)	<0.001
SXT (1.25/23.75 µg)	19 (20.4%)	1 (1.1%)	73 (78.5%)	70 (76.1%)	5 (5.4%)	17 (18.5%)	89 (48.1%)	6 (3.2%)	90 (48.6%)	<0.001
S (10 µg)	20 (21.5%)	15 (16.1%)	58 (62.4%)	55 (59.8%)	4 (4.3%)	33 (35.9%)	75 (40.5%)	19 (10.3%)	91 (49.2%)	<0.001

AK, amikacin; AM, ampicillin; AMC, amoxicillin/clavulanic acid; AZM, azithromycin; CAZ, ceftazidime; CIP, ciprofloxacin; C, chloramphenicol; CN, gentamicin; FOX, cefoxitin; CTX, cefotaxime; IPM, imipenem; NA, nalidixic acid; S, streptomycin; SXT, trimethoprim/sulfamethoxazole; TE, tetracycline; F, nitrofurantoin. Data are presented as *n* (%). R, resistant; I, intermediate; S, susceptible. Differences in susceptibility distributions (R/I/S) between beef and chicken isolates were assessed using Pearson’s chi-square test. Statistical significance was set at *p* < 0.05.

**Table 6:** Multiple antibiotic resistance (MAR) index of *E. coli* isolates (*n* = 185).

Number of Antibiotics	Beef Isolates *n* (%) (*n* = 93)	Chicken Isolates *n* (%) (*n* = 92)	Total Isolates *n* (%) (*n* = 185)	MAR Index
1	17 (18.3%)	1 (1.1%)	18 (9.7%)	0.06
2	8 (8.6%)	1 (1.1%)	9 (4.8%)	0.12
3	8 (8.6%)	5 (5.4%)	13 (7.0%)	0.18
4	7 (7.5%)	10 (10.9%)	17 (9.1%)	0.25
5	11 (11.8%)	17 (18.5%)	28 (15.1%)	0.31
6	1 (1.1%)	14 (15.2%)	15 (8.1%)	0.37
7	6 (6.5%)	5 (5.4%)	11 (5.9%)	0.43
8	2 (2.2%)	8 (8.7%)	10 (5.4%)	0.50
9	0 (0%)	14 (15.2%)	14 (7.5%)	0.56
10	3 (3.2%)	6 (6.5%)	9 (4.8%)	0.62
11	0 (0%)	6 (6.5%)	6 (3.2%)	0.68
12	0 (0%)	2 (2.2%)	2 (1.1%)	0.75
≥13	1 (1.1%)	0 (0%)	1 (0.5%)	1.00
Mean ± SD	0.165 ± 0.19	0.406 ± 0.18	0.285 ± 0.22	

Note: Thirty-two (17.2%) *E. coli* isolates showed no resistance to any of the tested antibiotics (MAR = 0.00). MAR indices were significantly higher in chicken isolates than in beef isolates (*p* < 0.001).

**Table 7:** Primers used in this study.

Primer	Primer Sequences (5′–3′)	Product Size (bp)	Annealing (°C)	Reference
*uspA*-F *uspA*-R	5′-CCGATACGCTGCCAATCAGT-3′ 5′-ACGCAGACCGTAGGCCAGAT-3′	884	63	Chen & Griffiths [40]
*stx1*-F *stx1*-R	5′-AGTTAATGTGGTGGCGAAGG-3′ 5′-CACCAGACAATGTAACCGC-3′	347	57	Fujioka et al. [41]
*stx2*-F *stx2*-R	5′-TTCGGTATCCTATTCCCGG-3′ 5′-CGTCATCGTATACACAGGAG-3′	589	57	Fujioka et al. [41]
*eae*-F *eae*-R	5′-GTGGCGAATACTGGCGAGACT-3′ 5′-CCCCATTCTTTTTCACCGTCG-3′	890	55	Gannon et al. [42]
*hlyA*-F *hlyA*-R	5′-ACGATGTGGTTTATTCTGGA-3′ 5′-CTTCACGTGACCATACATAT-3′	165	55	Fratamico et al. [43]

## Data Availability

The raw data supporting the conclusions of this article will be made available by the authors on request.

## Abbreviations

The following abbreviations are used in this manuscript:

AMR	Antimicrobial Resistance
ATCC	American Type Culture Collection
CFU	Colony-Forming Unit
CLSI	Clinical and Laboratory Standards Institute
EHEC	Enterohemorrhagic *Escherichia coli*
EMB	Eosin Methylene Blue Agar
HUS	Hemolytic Uremic Syndrome
MDR	Multidrug-Resistant
MAR	Multiple Antibiotic Resistance
MHA	Mueller–Hinton Agar
MHB	Mueller–Hinton Broth
STEC	Shiga Toxin-Producing *Escherichia coli*
TSA	Tryptic Soy Agar
TSB	Tryptic Soy Broth
VRBA	Violet Red Bile Agar
